# Editorial: *In vivo* and *in vitro* models for research in pathology

**DOI:** 10.3389/pore.2023.1611196

**Published:** 2023-04-04

**Authors:** Songwen Tan, Peter Nemeth

**Affiliations:** ^1^ Xiangya School of Pharmaceutical Sciences, Central South University, Changsha, China; ^2^ Department of Immunology and Biotechnology, University of Pécs, Medical School, Pécs, Hungary

**Keywords:** analytical pathology, pathogenesis, tumor diagnosis, *in vivo* techniques, molecular pathology

Analytical pathology is a critical research field for understanding the behavior of diseases in clinical applications (1, 2). Pathomorphological methods are utilized to examine pathological changes in organs, tissues, or cells of the body, which can aid in exploring the causes, pathogenesis, occurrence, and development of diseases and ultimately making a pathological diagnosis. Pathological examination of tumors is a vital method for tumor diagnosis and can identify the diagnosis, tissue origin, nature, and scope of tumors, providing an essential foundation for clinical treatment. *In vivo* pathological analysis involves various scanning techniques combined with mathematical analysis tools like computers and AI. Techniques such as ultrasound and nuclear magnetic resonance scanning (3), computed tomography (4), magnetic resonance imaging (5), and various other imaging technologies are used for *in vivo* pathological analysis. *In vitro* pathological analysis includes histochemistry, immunohistochemistry, molecular biology, and oncogene examination. With the rapid advancement of natural science, new instruments and technologies are being applied to medicine, such as ultrastructural pathology, molecular pathology, immunopathology, and genetic pathology (6).

In the field of pathology, *in vivo* and *in vitro* models have served as crucial tools for understanding the underlying mechanisms of diseases, as well as for the development of therapeutic strategies. The Special Issue “*In Vivo* and *In Vitro Models for Research in Pathology*” published in the Pathology and Oncology Research (POR) journal encompasses an assortment of scientific articles, highlighting recent advancements in various models for investigating diseases. This editorial paper provides a concise overview of five key publications within this Special Issue.

The first study (Wang et al.) provides important insights into the pathology of retinal ischemia reperfusion injury (RIRI) and the dynamic changes of the JAK-STAT signaling pathway and apoptosis in rats. The study highlights the importance of investigating the baselines at different time points when assessing the therapeutic effect of drugs, as the pathological changes and protein expression can vary over time. The results suggest that JAK-STAT signaling pathway activation plays a vital role in RIRI and that apoptosis is involved in the process, with a peak value at 24 h after injury. These findings suggest potential therapeutic directions and a time window for treating RIRI. Overall, the study provides valuable insights into the pathology and potential treatment of RIRI.

The second work (Jiang et al.) provides important insights into the role of KPNA1 in cervical cancer. The study highlights the significant association between KPNA1 expression levels and the histologic grade of cervical cancer, with lower expression levels observed in more malignant tumors. The study also reveals that upregulation of KPNA1 significantly suppresses the proliferation of Hela cells and promotes the transportation of IRF3 into the nucleus. These findings suggest that KPNA1 may play a critical role in the proliferation and progression of cervical cancer and may serve as a potential target for therapy. Overall, the study provides valuable insights into the pathogenesis of cervical cancer and highlights the importance of investigating the expression levels of KPNA proteins in different tumors.

The study on *Nocardia* rubra cell-wall skeleton (Nr-CWS) sheds light on its potential in cancer immunotherapy, particularly in enhancing natural killer (NK) cell function (Wu et al.). Although Nr-CWS demonstrated a limited impact on solid tumors, it significantly suppressed lung metastasis in a melanoma model, pointing to its ability to boost NK cell activity. The study further revealed that Nr-CWS upregulates the expression of vital NK cell markers and cytotoxic molecules, while promoting terminal differentiation. Consequently, the treated NK cells exhibited increased cytokine production and cytotoxicity. These findings suggest that Nr-CWS holds promise as a novel cancer immunotherapy component, warranting further investigation and potential clinical application in combating metastatic diseases.

Olanzapine, a second-generation antipsychotic drug, is effective in treating psychiatric illnesses but often causes metabolic side effects. This study investigated hyperbaric oxygen therapy (HBOT) as a potential approach to mitigate these side effects in rats (AlQudah et al.). The results showed that olanzapine treatment led to decreased serum insulin, triglyceride, and HDL cholesterol levels, while increasing fasting blood sugar and insulin resistance index. Interestingly, exposure to HBOT reversed these effects. Additionally, both HBOT and olanzapine upregulated pancreatic Langerhans islets, with the combined treatment doubling islet numbers. The study suggests that HBOT could be a promising alternative to manage olanzapine-associated metabolic disorders while positively affecting pancreatic Langerhans cells activity and architecture. Further research is needed to confirm these findings and assess the potential clinical applications of HBOT in patients treated with olanzapine.

The fifth paper (Sztankovics et al.) emphasizes the importance of 3D bioprinting in revolutionizing experimental cancer model systems, specifically in the context of *in vitro* 3D bioprinted breast cancer tissue-mimetic structures. Alternative technologies, such as human cell-based models or artificial intelligence-combined technologies, are increasingly important for accurately predicting human response and toxicity in medical research. The development of *in vitro* disease models aims to reduce and replace animal experiments, enabling more effective research, innovation, and drug testing. The authors present their newly established 3D bioprinted luminal B type breast cancer model, illustrating the advantages of these models in representing cancer tissue heterogeneity and *in vivo* conditions. However, the standardization of 3D bioprinting methods is crucial for future applications in high-throughput drug testing and patient-derived tumor models. The adoption of standardized bioprinted models could make cancer drug development more successful, efficient, and cost-effective in the future.

In conclusion, these studies collectively provide valuable insights into various aspects of medical research, from retinal ischemia reperfusion injury and cervical cancer pathogenesis to novel cancer immunotherapies and mitigating olanzapine-associated metabolic side effects. The importance of alternative technologies, such as 3D bioprinting, human cell-based models, and artificial intelligence, is underscored in the quest for more accurate predictions of human response and toxicity. While significant advancements have been made, challenges remain in standardizing methods and further investigating potential clinical applications. Continued research in these areas will not only contribute to a deeper understanding of disease pathogenesis but also pave the way for more effective, efficient, and cost-effective treatments in the future.
